# Students' intrinsic motivation in EFL academic writing: Topic-based interest in focus

**DOI:** 10.1016/j.heliyon.2024.e24169

**Published:** 2024-01-07

**Authors:** Ali Abbas Falah Alzubi, Mohd Nazim

**Affiliations:** Applied Linguistics, Department of English, College of Languages and Translation, Najran University, Kingdom of Saudi Arabia

**Keywords:** Topic-based interest, Intrinsic motivation, Attitudes, EFL writing context

## Abstract

Writing as a productive skill is extremely challenging for EFL students. To promote their writing effectiveness, students' intrinsic motivation must be enhanced by allowing them to self-assign writing topics or write about topics of interest. Therefore, this study describes how EFL students become motivated by self-assigning their writing topics and identifies students' attitudes and writing skills in topic-based interest in the EFL writing context. A descriptive–diagnostic method was applied to achieve the study objectives. A sample of 200 EFL students responded to a closed-item questionnaire on intrinsic motivation based on topic-based interest in writing. In addition, 20 students participated in semi-structured interviews on their attitudes toward topic-based interest. The study sample showed a high level of intrinsic motivation based on their self-assignment of writing topics. Furthermore, female students showed higher intrinsic motivation if they could self-assign their writing topics than male students. In addition, students with higher GPAs exhibited higher intrinsic motivation. Moreover, the interviews revealed that intrinsically motivated students most enhanced their writing skills in ideas, vocabulary, and choice of words. The researchers suggest that students' self-assignment of writing topics be considered in teaching writing as it contributes to improving intrinsic motivation.

## 1 Introduction

In the realm of EFL learning, intrinsic motivation characterizes a learner’s internal impetus and aspiration to engage in English language acquisition autonomously. This motivation is instrumental in cultivating a genuine and enduring affection for the English language. Learners endowed with intrinsic motivation exhibit a proclivity for self-directed learning, resilience in the face of challenges, and a heightened probability of attaining elevated levels of linguistic proficiency over time. Writing is considered the most difficult skill for EFL students because it requires them to employ various sub-skills and other elements, including grammar, choice of words, format, techniques, ideas, cohesion, coherence, and topic selection. One way to enhance students' writing skills is to promote their intrinsic motivation, through which they are internally pushed to produce more and write better. The existing research has extensively examined the issue of students' motivation in terms of their EFL writing performance, creativity, proficiency, engagement, achievement, and ability [[1], [2], [3], [4], [5], [6], [7], [8], [9], [10]]. In addition, research has been conducted on which intrinsic factors motivate students to write better; however, these internal factors, such as allowing students to self-assign their writing topics, have not been explored with Arabic-speaking learners. In addition, based on the researcher’s best knowledge, the relationship between students' self-assigned topics and intrinsic motivation and the impact of their GPAs and specializations (English, translation) has not been examined among Arabic-speaking learners. As such, the current study contributes to the existing body of knowledge in describing students' degree of intrinsic motivation in EFL academic writing. Additionally, it correlates students' text-based interest topics with their levels of intrinsic motivation. Moreover, the responses of the study sample are examined regarding students' gender, GPA, specialization, and study year. The following research questions respond to the study problem:

1. What is the degree of students' intrinsic motivation (topic interest) in EFL academic writing?
2. Do students' responses differ by gender, university degree, genre of writing, or GPA?
3. Which writing skills and genres correspond to students' text-based interest in the EFL academic writing context?

## 2 Literature review

### 2.1 Text-based interest in EFL academic writing

Interest is used to describe a range of intrinsic motivations and typically falls into one of two types: individual interest and situational interest. The individual interest perspective describes interest as a personal trait, an attraction emerging from experience, carried with the individual into different settings. Conversely, situational interest emerges from the situation. Interest, which results when an activity or topic has meaning for an individual [11], is an intrinsic motivator; engaging in an activity that is the subject of interest is inherently satisfying and requires no additional reward. Interest produces the tendency to pay attention to particular stimuli, partake in particular activities, and learn specific knowledge or abilities. In its connection with objects and stimuli, interest can be used in learning environment design to shape the set of objects learners interact with and the specific activities they undertake [12]. Interest is one of the motivational factors in writing [13], and individual interest in writing is likely to improve a writer’s motivation to write and make the writing process more enjoyable. People who have individual interests in topics tend to possess much topic knowledge about them. Teaching students to draw upon their interests for ideas is likely to have a similarly beneficial effect [14]. Ref. [15] state that topic attractiveness can be viewed as a basic motivational source of writing, an independent variable affecting the quality of the written text. Interest has tended to be viewed as rather static; students are thought to be either interested or uninterested in a particular topic they write about. Text-based interest thus can be seen as the first step toward interest in the topic. Topic interest is a reader’s cognitive and affective orientation to a topic, which leads them to perform autonomous operations, such as reflecting on, discussing, reading, and researching it [15]. Text-based interest originates in the salience of a text’s topics or themes or how the text is written. In addition, a text may be interesting in that it leads a reader to appreciate the topic’s relevance and search for new information relevant to it. Ref. [16] argues that students feel highly motivated when they are given the freedom to make choices about their learning process, while Ref. [17] discusses how the issue of self-selected materials is associated with the concept of choice-making: they can increase interest in learning a new language. Ref. [18] refer to how selecting well-organized texts can increase interest. Furthermore, Ref. [19] assert that topic selection in EFL writing can help learners explore the range of their vocabulary in producing language, which may otherwise not be utilized in teachers' assigned tasks. Ref. [20] explain that students are more motivated and encouraged to write when they are allowed to choose their topics in EFL writing classes, and Ref. [21] contend that topic selection enhances and motivates second-year English students' writing performance. Moreover, students claim that they prefer to write about topics they choose rather than those assigned to them. Finally, Ref. [22] maintain that offering students a choice in writing topics can lead to a higher fluency score as measured by a higher ratio of unique words to total words.

### 2.2 Motivation and writing skill

Much research has correlated motivation with EFL writing. Motivation has been extensively studied in relation to writing achievement [1], writing ability [2], L1 and L2 in- and out-of-school writing experiences [3], writing performance [[4], [5], [6]], success in L2 academic writing [7], writing proficiency [8], editing [9], and satisfaction and attitudes toward writing [10]. Prioritizing instructional methods that enhance the writing skills of students, all the while capitalizing on their intrinsic motivation for writing, is crucial, as it is an inherently gratifying and pleasurable endeavor. Ref. [6] investigated the writing performance and motivational beliefs of 880 students classified as English language learners by their school district. These students were tasked with composing an informative/explanatory essay on the topic of information technology and completed a motivational survey to gauge their intrinsic, extrinsic, and self-regulation motivations for writing. An overwhelming 97% of students did not achieve the expected grade-level proficiency in their writing, although writing scores tended to improve as students progressed through the six grade levels. A majority of the students acknowledged that both intrinsic and extrinsic motivators influenced their writing behavior, but only 38% felt that self-regulation motivations played a significant role. Furthermore, gender did not appear to be a factor in students' motivational scores. Engaging in creative writing exercises can serve as a motivational tool for learners, subsequently assisting in the enhancement of their writing and linguistic skills. Ref. [9] explored the impact of creative writing on enhancing the motivation of EFL learners to engage in writing activities at school and showed that creative writing played a significant role in stimulating EFL learners to generate written content. It appeared to foster a sense of significance for students by empowering writing endeavors that catered to their personal interests and revitalizing their perception of writing. To excel in writing, students must consistently nurture their motivation to participate in writing courses and to diligently tackle the assignments provided within these courses. Ref. [8] delved into the connection between motivation and writing skills among university students majoring in English. The students were required to compose essays and provide responses to a questionnaire assessing their motivation in writing. A significant correlation was revealed between the motivation levels of EFL students and their writing proficiency; students with higher levels of motivation in writing demonstrated superior writing proficiency. Additionally, the study highlighted that female students outshone their male counterparts in both motivation for writing and writing proficiency.

Writing performance correlates with motivation. Ref. [4] investigated the relationship between writing motivation and performance in students spanning grades three to five. The findings of this study, which utilized both a performance metric and a motivational assessment, revealed that emergent bilingual students and reclassified bilingual students exhibited significantly higher scores in intrinsic and self-regulatory motivation compared to their native English-speaking peers. Specifically, fourth graders scored significantly higher than third graders in terms of intrinsic motivation. Additionally, language status, gender, and grade influenced the variance in district writing scores and motivational incentives. Ref. [3] discourse-analyzed the literacy autobiographies of 25 participants to investigate whether there was a link between their motivation and writing in the school context. The results indicated that students' motivation for English academic writing was strongly connected to their academic writing in their native language. Ref. [2] investigated the connection between writing motivation and writing proficiency among high school students using a quantitative research approach. The results revealed a positive correlation between students' writing motivation and their writing ability. Ref. [1] delved into the effects on students' writing performance of their motivation to write, perceptions of the importance of writing achievement, and self-confidence as writers. Concentrating on middle and high school classrooms, the study cross-referenced data from students' writing samples, writing test scores, and teacher-assessed writing achievements and study established a significant and robust correlation between middle and high school students' motivation to write and their writing accomplishments.

Much research on students' intrinsic motivation in EFL academic writing exists. Enhancing self-efficacy beliefs and fostering intrinsic motivation in research can be achieved by providing students with options and independence, nurturing a sense of connection, and encouraging positive social collaboration. Ref. [23] conducted a study on self-efficacy beliefs and intrinsic motivation to assess undergraduate students' various research activities using a combination of tests and interviews. All measures showed a notable increase except for intrinsic motivation. In addition, a sense of connection or relatedness appeared to enhance intrinsic motivation for writing. Ref. [24] investigated students' perceptions regarding the factors influencing their ability to write descriptive essays using questionnaires and interviews. The results revealed that each student had unique factors that affected their writing process, such as low motivation, a limited vocabulary, a lack of knowledge in organizing ideas, and a diminished interest in the given topics. Likewise, Ref. [25] examined how motivated eleventh-grade high school students were in learning English writing. The study involved gathering information from students through questionnaires and conducting interviews with both students and their English teacher. The results showed that the students had a moderate level of motivation, combining both intrinsic and extrinsic factors. Specifically, the students' motivation was influenced by their high interest in the subject, their goals, and their curiosity. Similarly, Ref. [26] focused on investigating how schoolteachers view effective methods for inspiring students to engage in cross-curricular writing. The study employed various data collection techniques, including open-ended one-on-one interviews, classroom observations, and a focus group. The study’s findings revealed a set of best practices that teachers recommend for motivating students to write across different subjects, which include using captivating topics, creating personal connections, and allowing students to choose their own topics. Ref. [27] explored the perceptions of 18 high school students concerning essay writing, particularly when using standardized prompts generated by the teacher and prompts that involved students in developing the assignment. Data collection involved writing samples, surveys, and observations. Collectively, a majority of the students believed that they produced higher-quality work when the teacher generated the writing prompt. However, a slightly smaller number of students expressed a preference for the teacher–researcher to continue creating future writing prompts. Nonetheless, a significant portion of students still desired to be involved in the process of forming writing prompts. Topic attractiveness is an important factor for motivating teacher trainees to write outside the classroom. Ref. [28] investigated the motivations of 270 Turkish teacher trainees studying English to engage in English writing. An analysis of the data gathered through a questionnaire revealed that female participants were significantly motivated to write in English when the topic was appealing, while their male counterparts found motivation when teachers provided writing samples. Finally, interest can predict students' writing achievement. Ref. [29] examined the link between interest and performance in first year students' writing class through a questionnaire. The results showed high student interest in the writing class and journal writing. No gender differences were found in class interest, journal interest, or writing achievement. Additionally, class interest strongly predicted writing achievement.

## 3 Methodology

The study is descriptive in nature. It surveyed students' intrinsic motivation in the context of EFL academic writing and further diagnosed the relationship between students' intrinsic motivation (text-based interest topics) and their writing skills, performances, and genres. Finally, it correlated students' responses by their gender, university degree, and GPA.

### 3.1 Population and sample of the study

The study was conducted at a Saudi governmental university, among the population of students earning bachelor’s degrees in English or translation in the College of Languages and Translation. Only those students who had studied or were studying writing courses were involved in the study. The total population numbered 1000 students, who were Arabic-speaking learners of English, in the third semester of the 2023 academic year. The study sample was drawn randomly, and the first study instrument (a questionnaire) was designed electronically and then shared with the targeted groups of students through email, Blackboard, and WhatsApp. The number of students responding to the questionnaire reached 200 (20% of the population), and 20 of these participants also participated in interviews. The study was approved by the ethical approval committee at the university, and a consent form was collected from everyone who participated in the study. Table 1 presents the sample distribution based on gender, specialization, study year, and GPA.

**Table 1:** Sample distribution.

Variable	Group	No.	%
Gender	Male	108	54.0
	Female	92	46.0
Year	Year one	80	40.0
	Year two	46	23.0
	Year three	33	16.5
	Year four	41	20.5
GPA	>2	42	21.0
	2–2.99	51	25.5
	3–3.99	54	27.0
	4–5	53	26.5
Specialization	Translation	76	38.0
	English	124	62.0
**Total**		**200**	**100**

### 3.2 Instruments of the study

The study employed a closed-item questionnaire and a semi-structured interview. The questionnaire had three sections: an introduction, demographic variables, and intrinsic motivation (topic interest) in the context of EFL academic writing. The introduction contained information about the title and objective of the study, general instructions for respondents, and the consent statement for participation in the study, while the demographic part included information about the participants' gender, university degree, genre of writing, and GPA. Finally, the third part presented 20 statements about students' intrinsic motivation (topic interest) in EFL academic writing using a five-point Likert scale: strongly disagree–strongly agree. In addition, two statements examined students' writing skill performance and preferred genres when writing about a self-chosen topic. The questionnaire was developed based on existing research [8,30], which the researchers used to develop the questionnaire items and domains. To determine the degree of approval based on the range equation, the following grading was adopted for the degree of achievement of the items and domains of the study instrument: 1–1.80 = very low, >1.80–2.60 = low, >2.60–3.40 = medium, >3.40–4.20 = high, >4.20–5 = very high.

Semi-structured interviews were conducted with some participants, following their consent. The interviewees were asked questions concerning their experiences and feelings toward topic writing assignments if they self-assign the topic or write about a topic of their interest and to what degree this motivates them. The interviews were conducted online using Zoom and lasted an average of 5.32 min. All sessions were recorded and transcribed. The interviews were conducted in English, with Arabic prompts as needed.

#### 3.2.1 Validity and reliability

Face validity and internal consistency were applied to verify the validity of the study instruments: a questionnaire and a semi-structured interview. The face validity of the tools was checked by a jury of experts (N = 10) who specialize in English language teaching and learning. The questionnaire statements and the semi-structured interview were checked for their ability to measure the study objectives. In addition, the experts checked the wordiness and language of statements. The experts approved the tools and confirmed their ability to measure the study objectives. In addition, based on their suggestions and comments, the instruments were modified and subsequently rewritten in their final versions. Suggestions included rewriting certain items to ensure clarity, adding items, and replacing some words. Moreover, the study instruments were piloted to a sample of 20 students to verify their internal consistency using the Pearson correlation coefficient equation. Table 2 depicts the results.

**Table 2:** Results of internal consistency (questionnaire).

Item	Correlation Coefficient	Sig.	Item	Correlation Coefficient	Sig.
1	.741^a^	.000	11	.583^a^	.007
2	.575^a^	.008	12	.514*	.020
3	.452*	.045	13	.718^a^	.000
4	.575^a^	.008	14	.662^a^	.001
5	.650^a^	.002	15	.642^a^	.002
6	.650^a^	.002	16	.613^a^	.004
7	.593^a^	.006	17	.669^a^	.001
8	.753^a^	.000	18	.725^a^	.000
9	.704^a^	.003	19	.564^a^	.010
10	.729^a^	.000	20	.827^a^	.000

a Correlation is significant at the 0.01 level (2-tailed), *. Correlation is significant at the 0.05 level (2-tailed).

Table 2 shows that Pearson’s correlation coefficients between the items with the total score were statistically significant at 0.01 or 0.05 level. Furthermore, the correlation coefficients between the items with the score total for the scale ranged from 0.452 to 0.827. In addition, the split-half test (Guttman) was used to check the reliability coefficient values of the study instrument (questionnaire). According to the analysis results, the overall reliability coefficient was 0.86, indicating that the study tool enjoys very good reliability.

### 3.3 Data analysis

Both quantitative and qualitative analyses were used in this research. Descriptive statistics were used to analyze the data collected, including means, ranks, standard deviations, and inferential statistics: independent sample t-tests and analysis of variance (ANOVA). The data from the semi-structured interview were analyzed using content analysis, in which the data were sorted, read, and classified under specific themes.

## 4 Study results

### 4.1 Degree of students' intrinsic motivation (topic interest) in the EFL academic writing

Table 3 shows the means and standard deviations for the responses to the items of intrinsic motivation when they self-assign or write about a topic of interest.

**Table 3:** Descriptive statistics of students' intrinsic motivation.

No	Rank	Items	Means	Standard Deviation	Degree
1	17	I do better when I (myself) select a writing assignment topic	3.45	1.640	High
2	8	I use more creative and innovative ways to express when I write about a topic of my choice/interest	3.69	1.491	High
3	5	I enjoy writing about a topic of my choice/interest	3.78	1.370	High
4	2	I utilize my full potential when I write about a topic of my choice/interest	3.91	1.460	High
5	9	I write more extensively when I write about a topic of my choice/interest	3.68	1.406	High
6	10	I vary my word choice when I write about a topic of my choice/interest	3.68	1.493	High
7	20	I commit fewer mistakes when I write a topic of my choice/interest	3.22	1.542	Medium
8	19	I take less time when I write a topic of my choice/interest	3.32	1.490	Medium
9	14	My writing fluency is better when I write a topic of my choice/interest	3.53	1.566	High
10	16	My writing is more cohesive and coherent when I write a topic of my choice/interest	3.46	1.644	High
11	3	I feel more confident when I write a topic of my choice/interest	3.81	1.515	High
12	6	I feel more motivated when I write a topic of my choice/interest	3.69	1.412	High
13	18	I organize contents effectively when I write a topic of my choice/interest	3.35	1.519	High
14	1	I love writing about topics of my choice to express my inner self	3.96	1.435	High
15	11	I feel more controlled/responsible when I write a topic of my choice/interest	3.59	1.498	High
16	15	I am not bored when my teacher assigns me the writing topic	3.47	1.403	High
17	7	I immerse with my inherent satisfaction when I write a topic of my choice/interest	3.69	1.511	High
18	12	I move to act for the fun when I write a topic of my choice/interest	3.58	1.522	High
19	13	I sense self-directed when I write a topic of my choice/interest	3.53	1.337	High
20	4	I exhibit mastery when I write a topic of my choice/interest	3.79	1.351	High
		**Total**	**3.61**	**1.136**	**High**

Table 3 shows that the total score of students' intrinsic motivation for their self-assigned writing topic had a high degree (M = 3.61, SD = 1.13). This result means that the study sample enjoyed a high level of intrinsic motivation when given the chance by their teachers to self-assign their writing topic or write about a topic of their interest. In addition, the means of the questionnaire items ranged between (3.22–3.96). The participants' most highly intrinsic motivation was rated in expressing their inner self (M = 3.96, SD = 1.43), utilizing their full potential, (M = 3.91, SD = 1.46), being fully confident (M = 3.81, SD = 1.51) when writing about a topic of their choice or interest. However, they were least intrinsically motivated in the topic selection/interest in committing fewer mistakes (M = 3.22, SD = 1.54) and taking less time (M = 3.32, SD = 1.59).

### 4.2 The influence of gender, specialization, GPA, and study year

Table 4 shows the significance of the differences between the means of the responses of the study sample on the topic selection/interest in students' intrinsic motivation according to the variables of gender, specialization, GPA, and academic year. One-way analysis of variance was used to extract the results.

**Table 4:** One-way analysis for the sample’s responses based on their demographic variables.

Source	Group	No.	Mean	Std. Deviation	Type I Sum of Squares	df	Mean Square	F	Sig.
Gender	Male	108	3.25	1.146	30.217	1	30.217	31.360	.000
	Female	92	4.03	.973					
Specialization	Translation	76	3.78	.887	3.616	1	3.616	3.753	.054
	English	124	3.50	1.257					
GPA	4–5	53	4.10	.854	35.949	3	11.983	12.436	.000
	3-less than 4	54	3.79	1.152					
	2-less than 3	51	3.56	1.012					
	Less than 2	42	2.81	1.171					
Year	Year 1	80	3.42	1.253	3.198	3	1.066	1.106	.348
	Year 2	46	3.63	1.193					
	Year 3	33	3.72	.984					
	Year 4	41	3.86	.896					
Error					184.037	191	.964		
**Total**					**2861.632**	**200**			

Based on the results presented in Table 4, there was a notable disparity in the responses of the study sample depending on their gender, with females exhibiting a preference. The significance level of this difference, denoted as 0.000, was less than 0.01. This result indicates that the variable of gender indeed had an impact on the responses of the study sample. In addition, no statistically significant distinction was observed in the study sample’s responses with regard to their specialization, whether in Translation or English. This result suggests that the responses of the study sample remained consistent across these specializations. Moreover, the study did not reveal any statistically significant differences at the significance level of 0.05 concerning the study year. This result indicates that the responses of the study sample were not influenced by the specific study year, suggesting a lack of impact from the passage of time during the study.

Table 5 shows if statistically significant differences exist between the responses of the study sample at (0.05) according to the GPA variable. Multiple comparisons (Scheffe’s test) were used to show these differences.

**Table 5:** Multiple comparisons (Scheffes’s test) (GPA).

GPA (I)	GPA (J)	Mean Difference (I-J)	Sig.
4–5	3–3.99	.31	.433
	2–2.99	.55^a^	.048
	>2	1.29^a^	.000
3–3.99	4–5	−.31-	.433
	2–2.99	.23	.694
	>2	.97^a^	.000
2–2.99	4–5	−.55-a	.048
	3–3.99	−.23-	.694
	>2	.74^a^	.005

a (I) & (J) represent differences between multiple GPAs.

According to the data presented in Table 5, statistically significant differences were observed at the 0.05 significance level with respect to the GPA variable. These differences were evident between the GPA range of 4–5 and both of the other two GPA groups, specifically, less than 2–3 and less than 2, with the advantage leaning toward the GPA range of 4–5. Similarly, significant statistical differences were also found between the GPA range of 3–4 and the GPA group less than 2, favoring the 3–4 GPA range. Furthermore, distinctions were apparent between the GPA range of 2 - less than 3 and the GPA group less than 2, with the GPA range of 2 - less than 3 showing a difference.

### 4.3 Students' favored writing skills and genres and their text-based interest

**Fig. 1:**
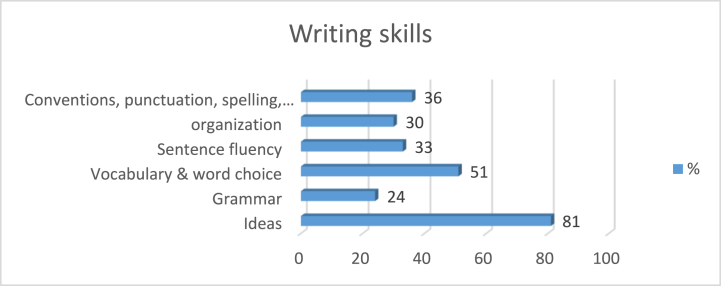
Writing skills and text-based interest.

Fig. 1 depicts the responses of the study sample to their favored writing skills and genres when they are intrinsically motivated by writing topic selection.

According to Fig. 1, students predominantly excel in writing skills, such as generating ideas, employing rich vocabulary, and making appropriate word choices when they select their own topics or write about subjects that pique their interest. In contrast, the skills of grammar and organization were least frequently demonstrated by the students.

**Fig. 2:**
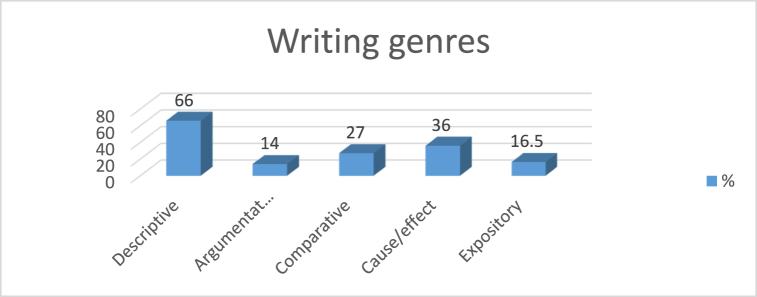
Writing genres and text-based interest.

In addition, Fig. 2 shows the writing genres that students like to write when they self-assign their writing topics.

In Fig. 2, it is evident that students preferred descriptive writing the most when they choose their own topics or write about subjects that interest them, while the least favored genre was argumentative writing.

### 4.4 Experiences and feelings about writing a topic of their interest or choice

The semi-structured interview targeted three main themes: reasons for feeling better when writing about a topic of a student’s choice, challenges, and barriers when the writing topic is of the teacher’s selection, and students' feelings when writing about what they like or are interested in. As stated earlier, the interview answers were transcribed, sorted, read, and reviewed. Then, they were classified under common topics and themes. Finally, the report was written along with some excerpts. Three themes have emerged in the first theme (reasons): fluency, information, and good feelings. Students stated that they are more fluent, less restricted, and freer when they write about a topic of their choice/interest. The following are some excerpts:

S4: I feel free to write in a manner that can make me improve as a writer

S9: Because this is something I wanted and loved, so with all confidence it makes me write better than writing a topic that is not of my choice.

S11: Because I have information about what I will write.

S18: When I’m writing I feel better more than I imagine.

S19: Write freely without restrictions, follow my mood, I am not bound by words, sentences come with my imagination automatically.

S20: I could express my feelings and thoughts fluency, and have more than one idea to write.

Regarding the second theme (challenges and barriers), four themes emerged: lack of information, time shortage, psychological factors, and writing errors. The interviewees explained that they do not have enough information, vocabulary, and ideas when they are not assigned their writing topics or of their interest as evident in the following excerpts:

S1: Perhaps I do not have enough information on the subject specified by the teacher.

S10: Difficulty finding words and arranging sentences in professional ways.

S20: When my teacher assigns me a topic to write about, I face a problem that I may not have enough words for the topic. I also suffer from a lack of ideas because I do not have more ideas for that topic.

Additionally, students hinted at the time issue that they do not have enough time when writing about a topic, not of their choice, and they have to search for information.

S2: time.

S4: It takes time to search for a topic that I don’t know much about.

Moreover, students indicated some psychological factors they feel when they are not assigned their writing topic selection, such as anxiety, fear, lack of focus, boredom, and laziness.

S3: I get scared and lack focus.

S19: Boredom and laziness.

However, the possibility of committing more writing errors was present in students' answers, such as spelling, grammar, punctuation, order or ideas, and choice of sentence.

S3: spelling and punctuation.

S10: arranging sentences in professional ways.

S16: grammatical mistakes.

S20: the mistakes of spelling.

As for the third theme (experiences and feelings), students, in general, liked the idea of writing about what they like as this makes them feel positive, achievement, and improvement. They feel satisfied, good, proud, confident, comfortable, and excited when writing about a topic of their choice/interest.

S1: I feel satisfied with my writing and more freedom to express what is on my mind.

S6: When I write, I feel that I am dispersing the energy that is in me, and I also feel that I enjoy writing, but some topics I don’t like, so I don’t want to write them.

S9: A beautiful and wonderful feeling when I felt that I had justified what was inside me in this matter.

S15: I feel free to express my thoughts and feelings about anything.

S18: Comfortable because I can express about myself.

S20: I felt so excited.

Besides, they feel that they have achieved something good and great.

S2: Nice feeling and accomplishment.

S4: I felt a sense of accomplishment after completing the task.

S7: Achievement.

S14: A sense of accomplishment and mastery.

In addition, they favored their writing skills, such as fluency, expression of ideas, and creativity.

S16: I like it as I can write fluently and more freely, despite the presence of grammatical errors, but it’s okay.

S18: I feel better at writing.

S19: A wonderful experience that put my creative fingerprints and spoke freely.

S20: I have confidence because I used my own style, innovative words and exceptional ideas.

## 5 Discussion

This study has examined the vital question of whether EFL students have a high level of intrinsic motivation when given the choice to self-select their writing topic or write about a topic of their interest.

### 5.1 What is the degree of students' intrinsic motivation (topic interest) in EFL academic writing?

The results indicate that study respondents showed a high level of intrinsic motivation based on their self-assignment of writing topics, which suggests that Arabic-speaking learners of English are likely more intrinsically motivated to perform better in terms of writing skills if their teacher allows them to write about a topic of their selection or interest. This result implies that for students to improve in writing, they must boost their intrinsic motivation by writing about topics of choice/interest. The result also aligns with previous research reporting that motivation in general, and intrinsic motivation in particular, is a dominant factor that influences success in learning EFL writing [1,13]. Topic attractiveness is a basic motivational source of writing that affects the quality of the written text [15]. The current result aligns with Ref. [4], who found that the students who had higher intrinsic and self-regulatory motivation scored better on a writing test. In addition, Ref. [3] revealed that students' motivation for English academic writing correlated with their L1 academic writing. According to Ref. [2], a link exists between students' writing motivation and ability, while Ref. [6] confirmed that intrinsic and extrinsic incentives could drive students' writing behavior.

### 5.2 Do students' responses differ by gender, university degree, genre of writing, or GPA?

Concerning the means of intrinsic motivation in the writing of Saudi EFL undergraduates across gender, specialization, GPA, and study year, female students showed a higher level of intrinsic motivation if they could self-assign their writing topics than did male students. This result is consistent with the results of previous research investigating the relationship between motivation and gender. Ref. [8] showed a link between motivation in writing and gender in favor of EFL female students in an Indonesian university, while Ref. [21] contend that topic selection enhances and motivates second-year English students' writing performance. In addition, Ref. [28] showed that the attractiveness of writing topics was an important motivating factor for female Turkish teachers. Conversely, Ref. [6] reported that gender did not relate to students' motivational scores in writing. Moreover, Ref. [29] found no difference between male and female students in their interest in their writing class, interest in journal writing, or writing achievement. The contrasting results may be attributed to differences in study context, language background, and students' specialization and year.

A comparison of the results of the means of the intrinsic motivation of Saudi EFL undergraduates according to their GPA revealed that the students who had higher GPAs exhibited a higher level of intrinsic motivation in writing about a topic of their selection/interest. This implies that students' intrinsic motivation is connected to their GPA; if students have a high GPA, they tend to show a high level of intrinsic motivation. This result aligns with existing research. Ref. [8] reported that students who showed a higher level of motivation performed better in their writing proficiency, while Ref. [17] showed that topic selection impacted EFL students' writing performance. Finally, Ref. [1] demonstrated that students' motivation correlated highly with their writing achievement.

With regard to the results of the mean comparison of EFL students' levels of intrinsic motivation with their specialization, the responses of the study sample did not vary by specialization (English/translation). This result means that no link was found between students' level of intrinsic motivation and their specialization, which may be explained by the fact that writing is a language skill that students in almost all majors of English must develop; therefore, it specialization was not expected to affect the responses of the study sample. Additionally, the students' study year was not found to be significant; it did not affect students' levels of intrinsic motivation when selecting writing topics. These results contribute to the existing body of research in providing details on the nature of the relationship between students' specialization and study year and their level of intrinsic motivation in the EFL writing context.

In addition, students' intrinsic motivation (topic-based interest) was correlated with improvements in their writing skills. The results showed that students who were intrinsically motivated most enhanced their writing skills in ideas, vocabulary, and choice of words. Ref. [19] assert that topic selection in EFL writing can help learners explore the range of their vocabulary in producing language. Furthermore, students liked the descriptive genre of writing if self-assigned their writing topics. This result suggests that students prefer the descriptive genre of writing when writing about a topic of their choice.

### 5.3 Which writing skills and genres correspond to students' text-based interest in the EFL academic writing context?

The content analysis of the data from the semi-structured interviews targeted three main aspects: reasons for feeling better when writing about a topic of a student’s choice, challenges and barriers for writing about a topic of the teacher’s selection, and students' feelings when writing about what they liked or were interested in. Students' feeling better when writing about a topic of their choice or interest was due to their being fluent in writing, possessing sufficient information, and having good feelings. Students were more fluent, less restricted, and freer when writing about a topic of their choice/interest. The challenges and barriers for writing about a topic of the teacher’s choice revolved around a lack of information; time constraints; psychological factors, such as anxiety, fear, lack of focus, boredom, and laziness; and writing errors, such as spelling, grammar, punctuation, order of ideas, and choice of sentences. In addition, students, in general, liked the idea of writing about what they liked, as this makes them feel positive, achieved, and improved: they felt satisfied, good, proud, confident, comfortable, and excited when writing about topics of their choice/interest. Moreover, they felt that they had achieved something great. Furthermore, they favored their writing skills, such as fluency, expression of ideas, and creativity. Ref. [22] argue that when students have a choice in writing topics, they show a higher level of fluency. This result is partially consistent with Ref. [31], who indicate that high-interest topics facilitated better-quality ideas.

## 6 Implications

In the realm of educational policy and practice, the introduction of student-driven topic selection for writing assignments can yield profound implications for student motivation and educational outcomes. This pedagogical approach, characterized by student autonomy and choice-based learning, can have a multitude of consequential effects. Firstly, granting students the autonomy to select topics that align with their personal interests fosters a heightened level of enthusiasm and dedication. This intrinsic motivation results in heightened engagement, as students are more inclined to invest their time and effort in subjects that resonate with their passions. Furthermore, writing about topics that personally interest students enhances the relevance of their education to their daily lives. They can discern the practical applications of their learning, thus kindling their curiosity and motivation. This bridge between the classroom and the real world infuses education with greater meaning. The element of choice empowers students, providing them with a sense of ownership over their educational journey. They learn how to make decisions, set objectives, and assume responsibility for their work, contributing to the development of their autonomy, self-esteem, and self-efficacy. The impact also extends to the diversity of perspectives and interests within the classroom. When students are encouraged to pursue topics they are passionate about, it diversifies the range of subjects explored. This diversity broadens the horizons of both educators and fellow students, creating a richer, more inclusive learning environment. Critical thinking and problem-solving skills are further honed when students write about subjects they are passionate about. They delve into complex issues, analyze diverse viewpoints, and achieve a profound understanding of their chosen subjects. Additionally, the approach caters to the uniqueness of individual students—each with varying strengths, interests, and learning styles. Allowing students to select their own topics promotes a personalized and tailored learning experience, facilitating the realization of each student’s full potential. The implications are not confined to the immediate academic setting. Encouraging students to explore topics of their choosing may instill a lifelong love for learning, extending beyond the classroom. This passion for self-improvement can drive students to become dedicated, lifelong learners. Writing about topics of personal interest typically results in more refined and meaningful work. The motivation and enthusiasm students bring to their writing often lead to the production of higher-quality assignments. Additionally, the teacher–student relationship benefits from this approach. Teachers gain a deeper understanding of their students' interests, which can create a more supportive and positive learning environment. This increased understanding can also help teachers tailor their instruction to better meet the needs and interests of their students.

## 7 Conclusion

This study has examined how students' topic-based interest influences their intrinsic motivation, attitudes, and writing skills. The results indicate that study respondents showed a high level of intrinsic motivation based on their self-assignment of writing topics. Furthermore, female students showed higher intrinsic motivation if they could self-assign their writing topics, and students with higher GPAs exhibited higher intrinsic motivation; however, the responses of the study sample did not vary according to specialization (English/translation). Furthermore, the results reveal that the students who were intrinsically motivated primarily enhanced their writing skills in ideas, vocabulary, and choice of words and liked the descriptive genre of writing if self-assigning their writing topics. This study contributes to the existing research in providing evidence of the role of EFL students' topic selection in raising their intrinsic motivation and thus improving their writing skills. Additionally, it implies that teachers should allow their students to choose their writing topic or write about a topic of interest because this would increase their internal motivation and thus improve their writing skills, including ideas, creativity, vocabulary, and mechanisms. The current study focused only on EFL students' intrinsic motivation; the researchers developed a questionnaire on topic-based interest as one factor that can boost students' inner motivations in the EFL writing context. Future studies may use the questionnaire in other contexts, such as Western Asian countries. In addition, experimental studies on the link between students' topic-based interest and writing performance are recommended. The generalization of the current findings is limited by the topic examined (intrinsic motivation through topic-based interest). Moreover, the study context (EFL writing skills among students majoring English/translation) may limit the generalizability of the findings. Finally, the generalization of findings may be governed by the psychometrics of reliability and validity of the questionnaire.

## Data availability statement

The authors cannot to share the data as the participants have explicitly requested that it remains confidential and not be disclosed.

## Ethics statement

The approval letter for conducting the research from the Ethical Approval Committee at the Deanship of Scientific Research, Najran University was obtained with the code [010289-022558-DS]. A written informed consent form was also collected from each participant.

## CRediT authorship contribution statement

**Ali Abbas Falah Alzubi:** Writing - review & editing, Writing - original draft, Visualization, Validation, Supervision, Software, Resources, Project administration, Methodology, Investigation, Funding acquisition, Formal analysis, Data curation, Conceptualization. **Mohd Nazim:** Writing - review & editing, Writing - original draft, Visualization, Validation, Supervision, Software, Resources, Project administration, Methodology, Investigation, Data curation, Conceptualization.

## Declaration of competing interest

The authors declare the following financial interests/personal relationships which may be considered as potential competing interests: Ali Abbas Falah Alzubi reports a relationship with 10.13039/501100005911Najran University that includes: funding grants. If there are other authors, they declare that they have no known competing financial interests or personal relationships that could have appeared to influence the work reported in this paper.
